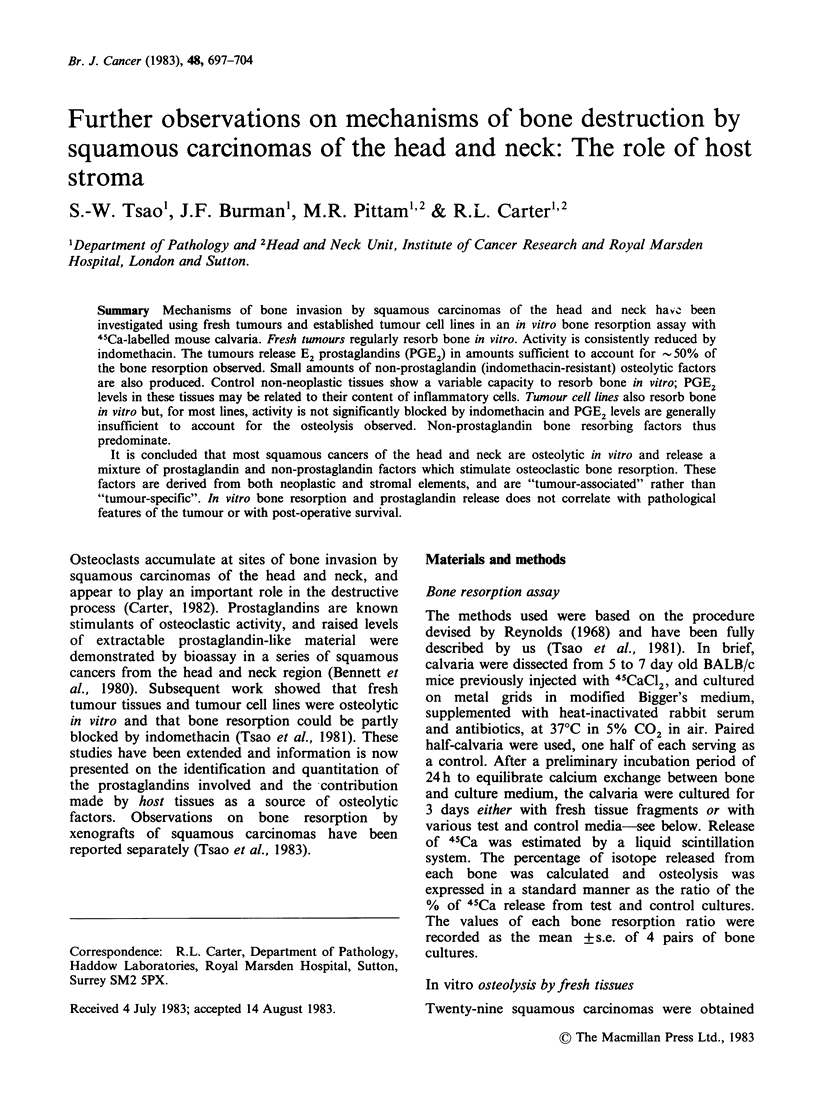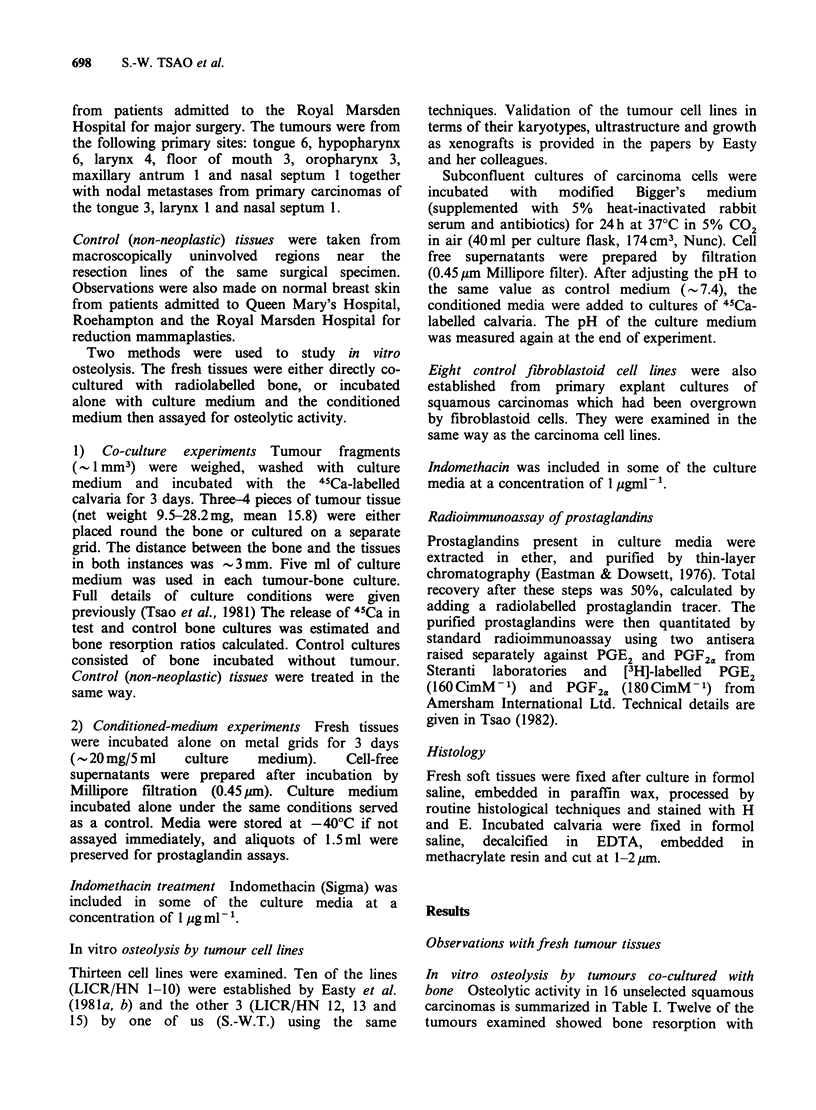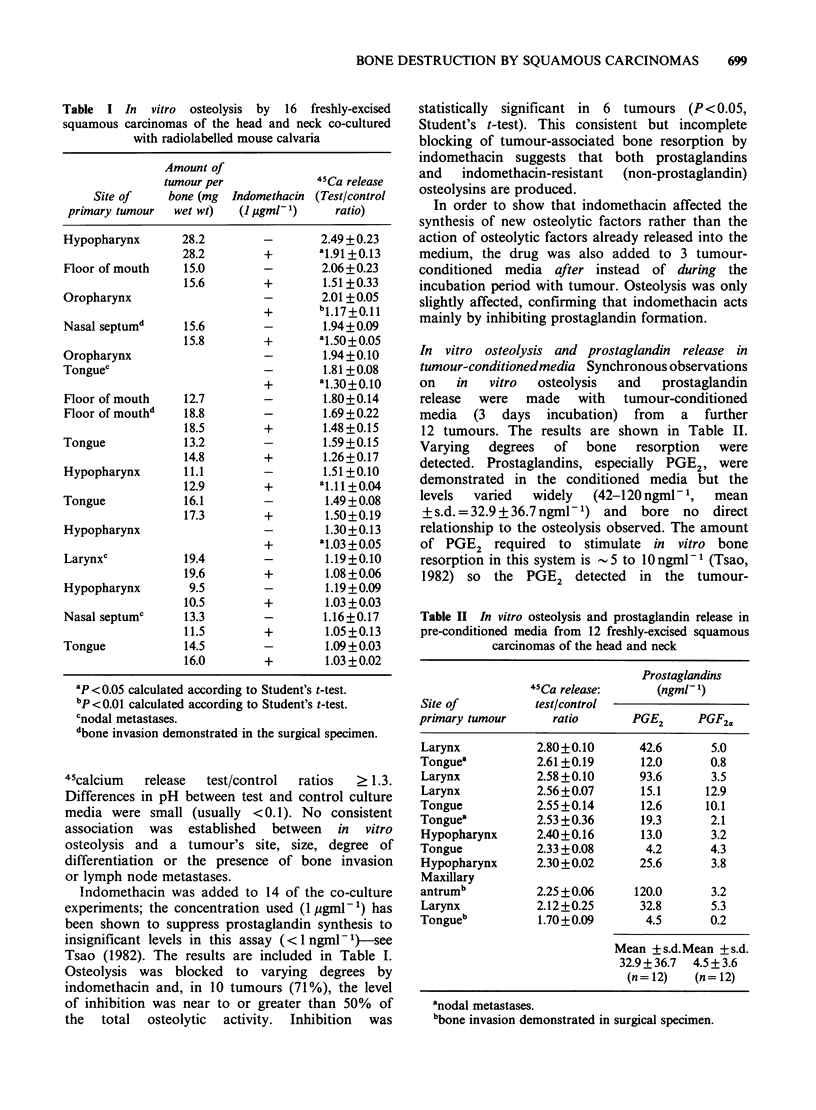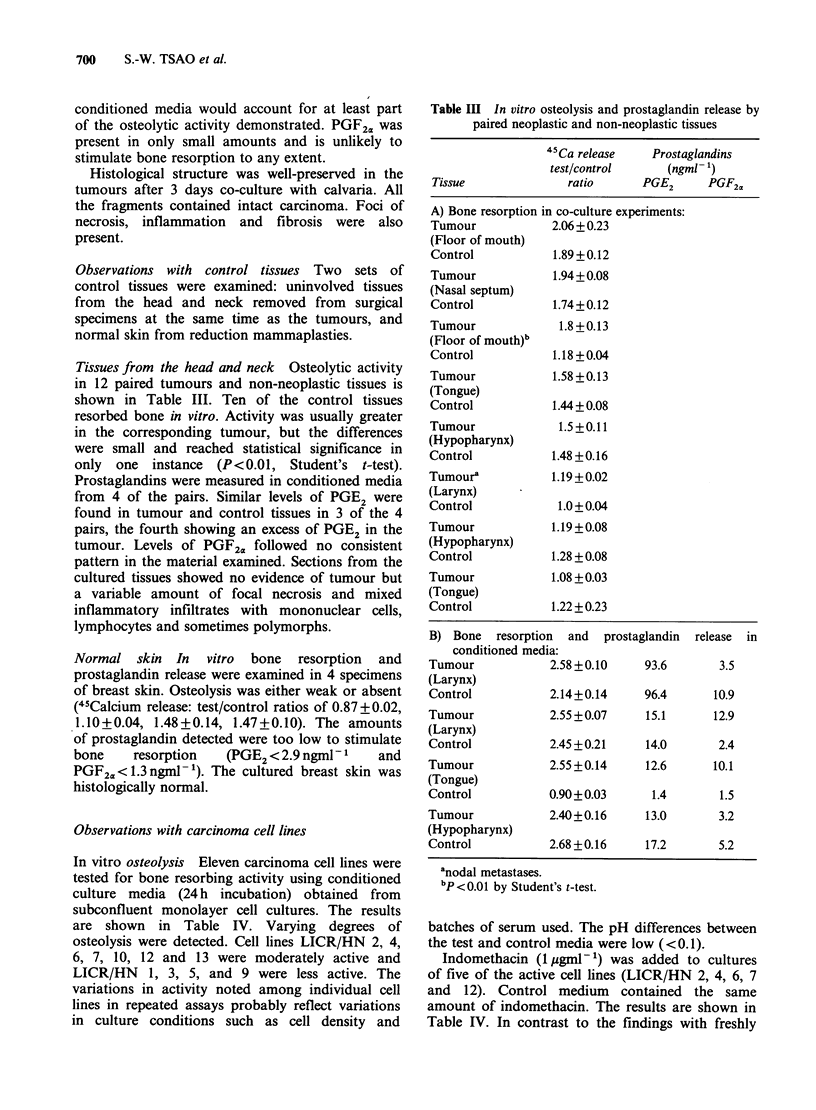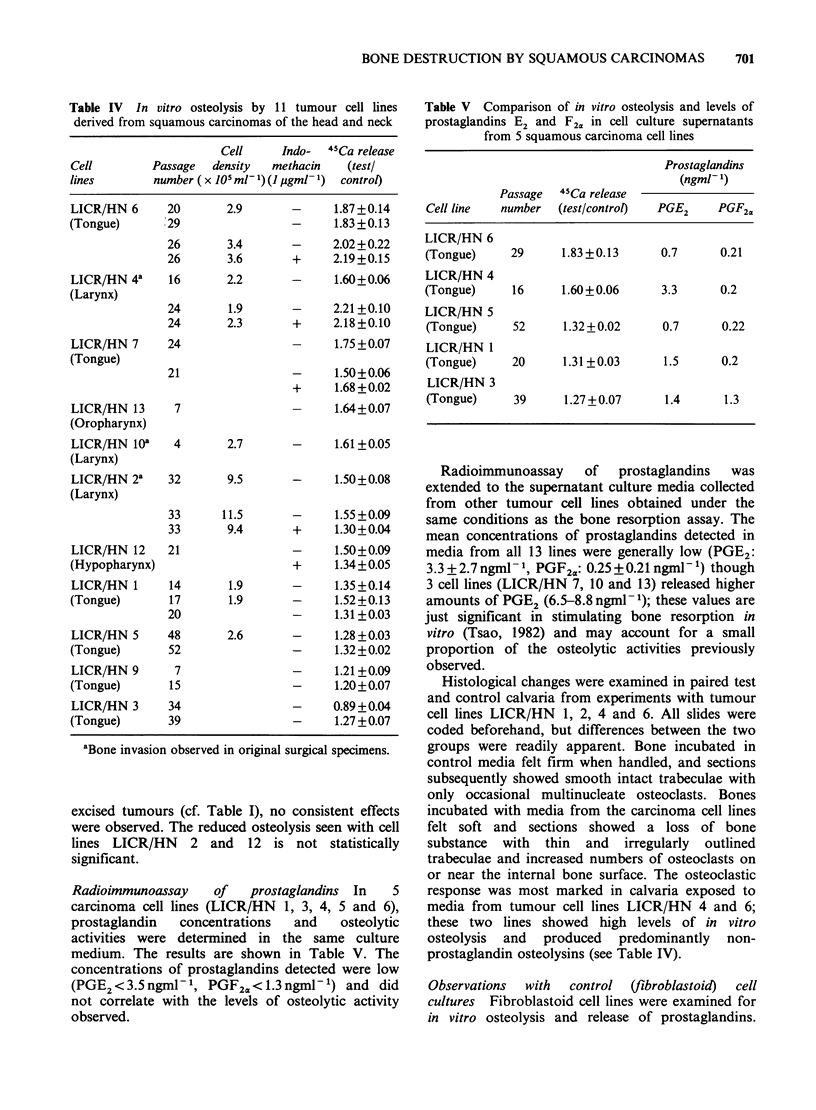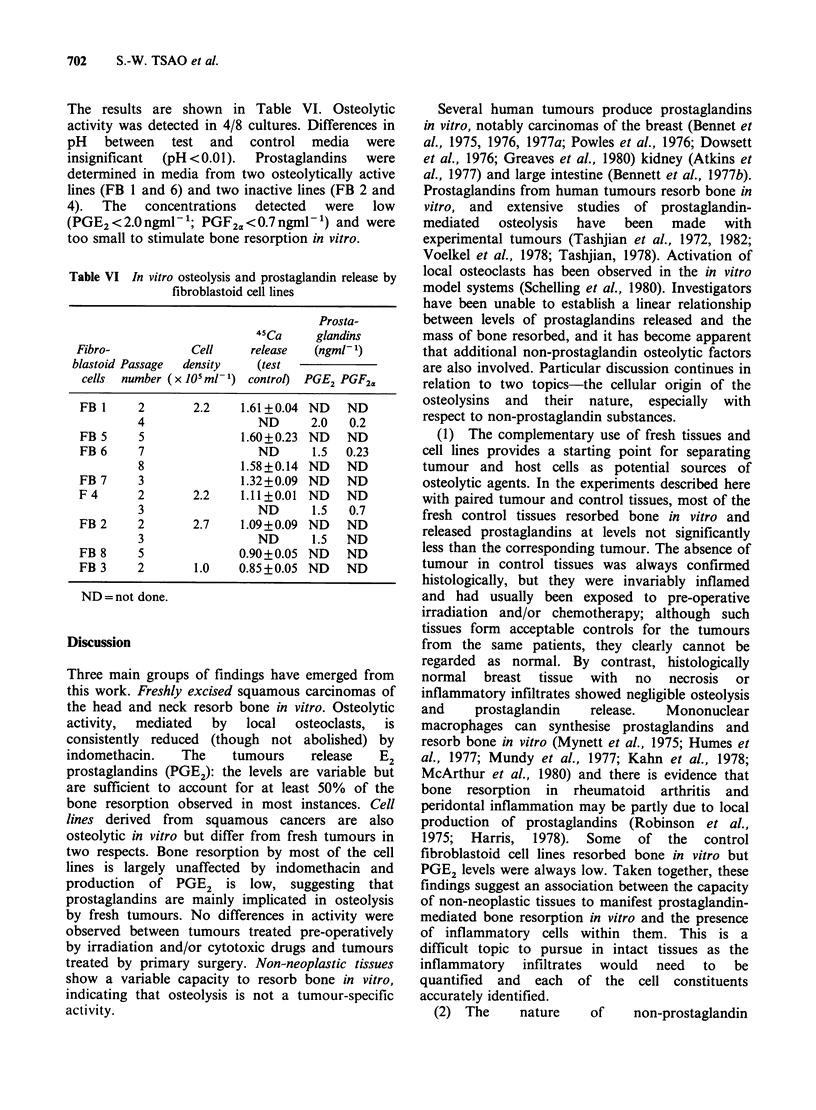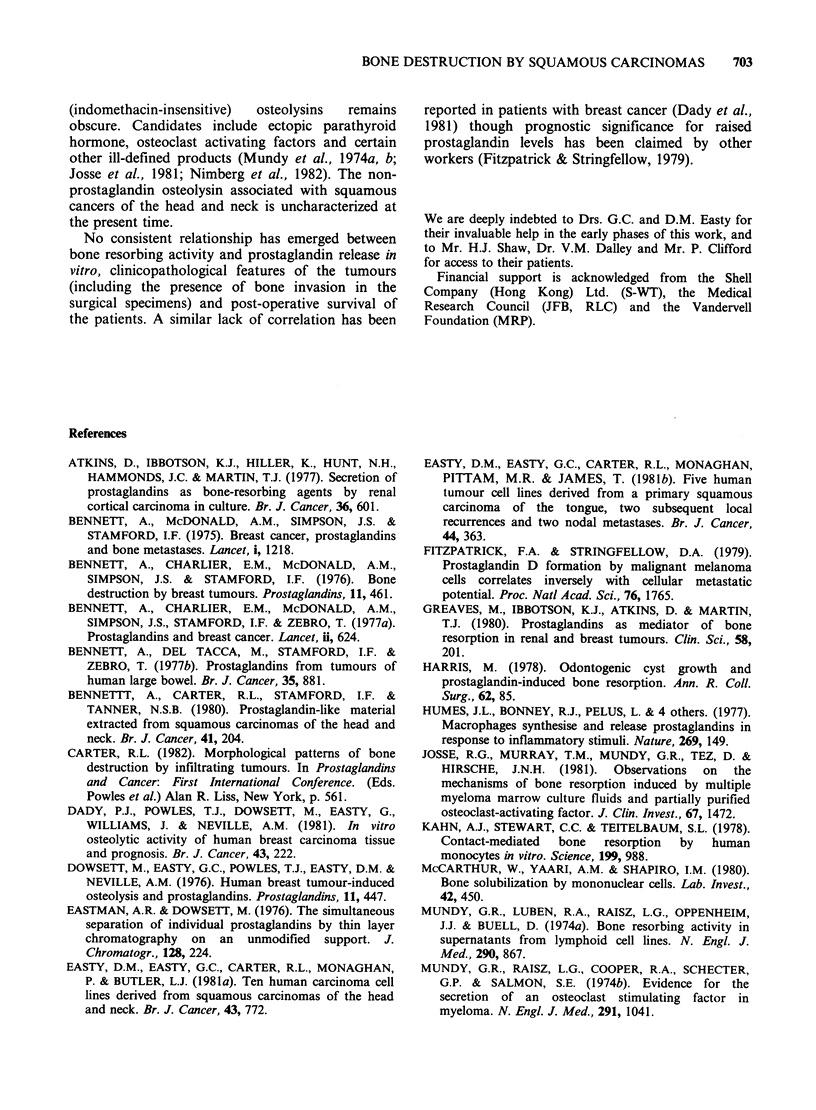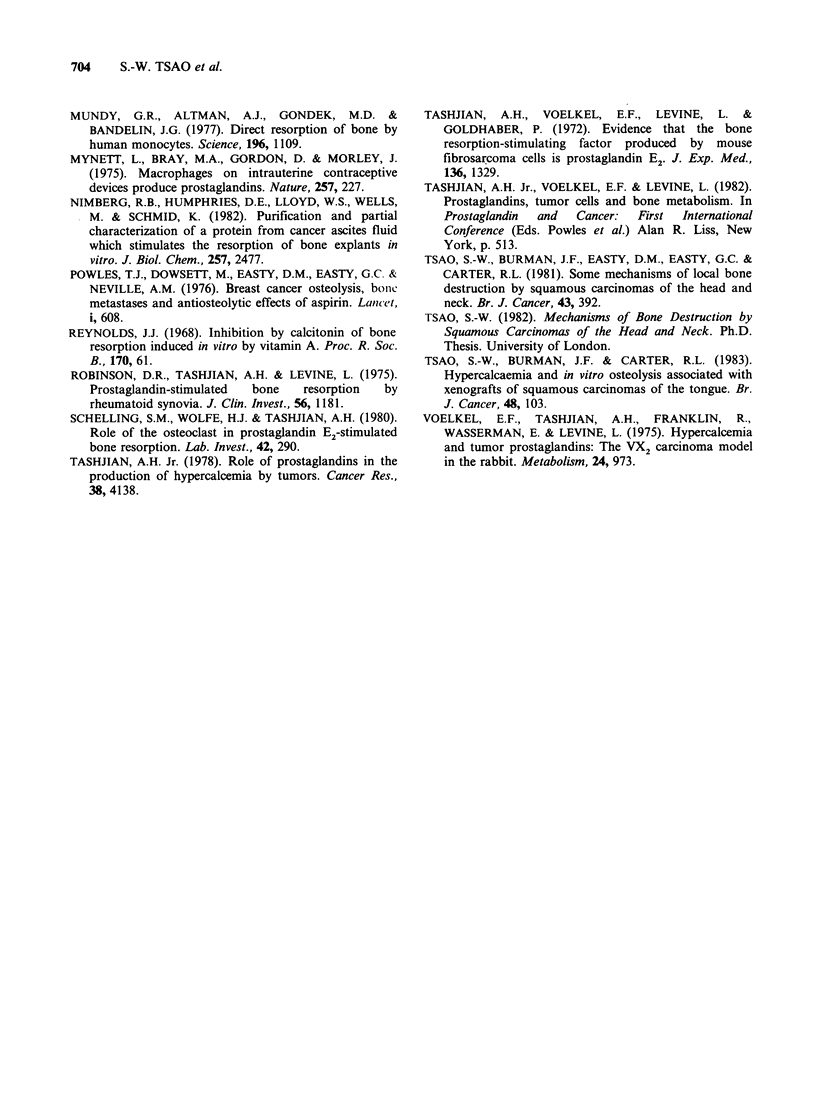# Further observations on mechanisms of bone destruction by squamous carcinomas of the head and neck: the role of host stroma.

**DOI:** 10.1038/bjc.1983.252

**Published:** 1983-11

**Authors:** S. W. Tsao, J. F. Burman, M. R. Pittam, R. L. Carter

## Abstract

Mechanisms of bone invasion by squamous carcinomas of the head and neck have been investigated using fresh tumours and established tumour cell lines in an in vitro bone resorption assay with 45Ca-labelled mouse calvaria. Fresh tumours regularly resorb bone in vitro. Activity is consistently reduced by indomethacin. The tumours release E2 prostaglandins (PGE2) in amounts sufficient to account for approximately 50% of the bone resorption observed. Small amounts of non-prostaglandin (indomethacin-resistant) osteolytic factors are also produced. Control non-neoplastic tissues show a variable capacity to resorb bone in vitro; PGE2 levels in these tissues may be related to their content of inflammatory cells. Tumour cell lines also resorb bone in vitro but, for most lines, activity is not significantly blocked by indomethacin and PGE2 levels are generally insufficient to account for the osteolysis observed. Non-prostaglandin bone resorbing factors thus predominate. It is concluded that most squamous cancers of the head and neck are osteolytic in vitro and release a mixture of prostaglandin and non-prostaglandin factors which stimulate osteoclastic bone resorption. These factors are derived from both neoplastic and stromal elements, and are "tumour-associated" rather than "tumour-specific". In vitro bone resorption and prostaglandin release does not correlate with pathological features of the tumour or with post-operative survival.


*Br. J. Cancer* (1983), **48**, 697–704

### Further observations on mechanisms of bone destruction by squamous carcinomas of the head and neck: The role of host stroma

S.-W. Tsao[1], J.F. Burman[1], M.R. Pittam[1,2] & R.L. Carter[1,2]

[1]*Department of Pathology and* [2]*Head and Neck Unit, Institute of Cancer Research and Royal Marsden Hospital, London and Sutton.*

**Summary** Mechanisms of bone invasion by squamous carcinomas of the head and neck have been investigated using fresh tumours and established tumour cell lines in an *in vitro* bone resorption assay with $^{45}Ca$-labelled mouse calvaria. *Fresh tumours* regularly resorb bone *in vitro*. Activity is consistently reduced by indomethacin. The tumours release $E_2$ prostaglandins ($PGE_2$) in amounts sufficient to account for ~50% of the bone resorption observed. Small amounts of non-prostaglandin (indomethacin-resistant) osteolytic factors are also produced. Control non-neoplastic tissues show a variable capacity to resorb bone *in vitro*; $PGE_2$ levels in these tissues may be related to their content of inflammatory cells. *Tumour cell lines* also resorb bone *in vitro* but, for most lines, activity is not significantly blocked by indomethacin and $PGE_2$ levels are generally insufficient to account for the osteolysis observed. Non-prostaglandin bone resorbing factors thus predominate.

It is concluded that most squamous cancers of the head and neck are osteolytic *in vitro* and release a mixture of prostaglandin and non-prostaglandin factors which stimulate osteoclastic bone resorption. These factors are derived from both neoplastic and stromal elements, and are "tumour-associated" rather than "tumour-specific". *In vitro* bone resorption and prostaglandin release does not correlate with pathological features of the tumour or with post-operative survival.

Osteoclasts accumulate at sites of bone invasion by squamous carcinomas of the head and neck, and appear to play an important role in the destructive process (Carter, 1982). Prostaglandins are known stimulants of osteoclastic activity, and raised levels of extractable prostaglandin-like material were demonstrated by bioassay in a series of squamous cancers from the head and neck region (Bennett *et al.,* 1980). Subsequent work showed that fresh tumour tissues and tumour cell lines were osteolytic *in vitro* and that bone resorption could be partly blocked by indomethacin (Tsao *et al.,* 1981). These studies have been extended and information is now presented on the identification and quantitation of the prostaglandins involved and the contribution made by *host* tissues as a source of osteolytic factors. Observations on bone resorption by xenografts of squamous carcinomas have been reported separately (Tsao *et al.,* 1983).

Correspondence: R.L. Carter, Department of Pathology, Haddow Laboratories, Royal Marsden Hospital, Sutton, Surrey SM2 5PX.

Received 4 July 1983; accepted 14 August 1983.

### Materials and methods

### Bone resorption assay

The methods used were based on the procedure devised by Reynolds (1968) and have been fully described by us (Tsao *et al.,* 1981). In brief, calvaria were dissected from 5 to 7 day old BALB/c mice previously injected with $^{45}CaCl_2$, and cultured on metal grids in modified Bigger's medium, supplemented with heat-inactivated rabbit serum and antibiotics, at 37°C in 5% $CO_2$ in air. Paired half-calvaria were used, one half of each serving as a control. After a preliminary incubation period of 24 h to equilibrate calcium exchange between bone and culture medium, the calvaria were cultured for 3 days *either* with fresh tissue fragments *or* with various test and control media—see below. Release of $^{45}Ca$ was estimated by a liquid scintillation system. The percentage of isotope released from each bone was calculated and osteolysis was expressed in a standard manner as the ratio of the % of $^{45}Ca$ release from test and control cultures. The values of each bone resorption ratio were recorded as the mean ±s.e. of 4 pairs of bone cultures.

### In vitro *osteolysis by fresh tissues*

Twenty-nine squamous carcinomas were obtained

© The Macmillan Press Ltd., 1983

from patients admitted to the Royal Marsden Hospital for major surgery. The tumours were from the following primary sites: tongue 6, hypopharynx 6, larynx 4, floor of mouth 3, oropharynx 3, maxillary antrum 1 and nasal septum 1 together with nodal metastases from primary carcinomas of the tongue 3, larynx 1 and nasal septum 1.

*Control (non-neoplastic) tissues* were taken from macroscopically uninvolved regions near the resection lines of the same surgical specimen. Observations were also made on normal breast skin from patients admitted to Queen Mary's Hospital, Roehampton and the Royal Marsden Hospital for reduction mammaplasties.

Two methods were used to study *in vitro* osteolysis. The fresh tissues were either directly co-cultured with radiolabelled bone, or incubated alone with culture medium and the conditioned medium then assayed for osteolytic activity.

1) *Co-culture experiments* Tumour fragments ($\sim 1\,mm^3$) were weighed, washed with culture medium and incubated with the $^{45}Ca$-labelled calvaria for 3 days. Three–4 pieces of tumour tissue (net weight 9.5–28.2 mg, mean 15.8) were either placed round the bone or cultured on a separate grid. The distance between the bone and the tissues in both instances was $\sim 3$ mm. Five ml of culture medium was used in each tumour-bone culture. Full details of culture conditions were given previously (Tsao *et al.*, 1981) The release of $^{45}Ca$ in test and control bone cultures was estimated and bone resorption ratios calculated. Control cultures consisted of bone incubated without tumour. *Control (non-neoplastic) tissues* were treated in the same way.

2) *Conditioned-medium experiments* Fresh tissues were incubated alone on metal grids for 3 days ($\sim 20$ mg/5 ml culture medium). Cell-free supernatants were prepared after incubation by Millipore filtration (0.45 $\mu$m). Culture medium incubated alone under the same conditions served as a control. Media were stored at $-40°C$ if not assayed immediately, and aliquots of 1.5 ml were preserved for prostaglandin assays.

*Indomethacin treatment* Indomethacin (Sigma) was included in some of the culture media at a concentration of $1\,\mu g\,ml^{-1}$.

### In vitro *osteolysis by tumour cell lines*

Thirteen cell lines were examined. Ten of the lines (LICR/HN 1–10) were established by Easty *et al.* (1981*a, b*) and the other 3 (LICR/HN 12, 13 and 15) by one of us (S.-W.T.) using the same techniques. Validation of the tumour cell lines in terms of their karyotypes, ultrastructure and growth as xenografts is provided in the papers by Easty and her colleagues.

Subconfluent cultures of carcinoma cells were incubated with modified Bigger's medium (supplemented with 5% heat-inactivated rabbit serum and antibiotics) for 24 h at 37°C in 5% $CO_2$ in air (40 ml per culture flask, 174 $cm^3$, Nunc). Cell free supernatants were prepared by filtration (0.45 $\mu$m Millipore filter). After adjusting the pH to the same value as control medium ($\sim 7.4$), the conditioned media were added to cultures of $^{45}Ca$-labelled calvaria. The pH of the culture medium was measured again at the end of experiment.

*Eight control fibroblastoid cell lines* were also established from primary explant cultures of squamous carcinomas which had been overgrown by fibroblastoid cells. They were examined in the same way as the carcinoma cell lines.

*Indomethacin* was included in some of the culture media at a concentration of $1\,\mu g ml^{-1}$.

### *Radioimmunoassay of prostaglandins*

Prostaglandins present in culture media were extracted in ether, and purified by thin-layer chromatography (Eastman & Dowsett, 1976). Total recovery after these steps was 50%, calculated by adding a radiolabelled prostaglandin tracer. The purified prostaglandins were then quantitated by standard radioimmunoassay using two antisera raised separately against $PGE_2$ and $PGF_{2\alpha}$ from Steranti laboratories and [$^3H$]-labelled $PGE_2$ ($160\,CimM^{-1}$) and $PGF_{2\alpha}$ ($180\,CimM^{-1}$) from Amersham International Ltd. Technical details are given in Tsao (1982).

### *Histology*

Fresh soft tissues were fixed after culture in formol saline, embedded in paraffin wax, processed by routine histological techniques and stained with H and E. Incubated calvaria were fixed in formol saline, decalcified in EDTA, embedded in methacrylate resin and cut at 1–2 $\mu$m.

### Results

### *Observations with fresh tumour tissues*

*In vitro osteolysis by tumours co-cultured with bone* Osteolytic activity in 16 unselected squamous carcinomas is summarized in Table I. Twelve of the tumours examined showed bone resorption with

**Table I** *In vitro* osteolysis by 16 freshly-excised squamous carcinomas of the head and neck co-cultured with radiolabelled mouse calvaria

| *Site of primary tumour* | *Amount of tumour per bone (mg wet wt)* | *Indomethacin ($1\ \mu gml^{-1}$)* | *$^{45}Ca$ release (Test/control ratio)* |
|---|---|---|---|
| Hypopharynx | 28.2 | – | 2.49±0.23 |
| | 28.2 | + | [a]1.91±0.13 |
| Floor of mouth | 15.0 | – | 2.06±0.23 |
| | 15.6 | + | 1.51±0.33 |
| Oropharynx | | – | 2.01±0.05 |
| | | + | [b]1.17±0.11 |
| Nasal septum[d] | 15.6 | – | 1.94±0.09 |
| | 15.8 | + | [a]1.50±0.05 |
| Oropharynx | | – | 1.94±0.10 |
| Tongue[c] | | – | 1.81±0.08 |
| | | + | [a]1.30±0.10 |
| Floor of mouth | 12.7 | – | 1.80±0.14 |
| Floor of mouth[d] | 18.8 | – | 1.69±0.22 |
| | 18.5 | + | 1.48±0.15 |
| Tongue | 13.2 | – | 1.59±0.15 |
| | 14.8 | + | 1.26±0.17 |
| Hypopharynx | 11.1 | – | 1.51±0.10 |
| | 12.9 | + | [a]1.11±0.04 |
| Tongue | 16.1 | – | 1.49±0.08 |
| | 17.3 | + | 1.50±0.19 |
| Hypopharynx | | – | 1.30±0.13 |
| | | + | [a]1.03±0.05 |
| Larynx[c] | 19.4 | – | 1.19±0.10 |
| | 19.6 | + | 1.08±0.06 |
| Hypopharynx | 9.5 | – | 1.19±0.09 |
| | 10.5 | + | 1.03±0.03 |
| Nasal septum[c] | 13.3 | – | 1.16±0.17 |
| | 11.5 | + | 1.05±0.13 |
| Tongue | 14.5 | – | 1.09±0.03 |
| | 16.0 | + | 1.03±0.02 |

[a]$P<0.05$ calculated according to Student's *t*-test.
[b]$P<0.01$ calculated according to Student's *t*-test.
[c]nodal metastases.
[d]bone invasion demonstrated in the surgical specimen.

$^{45}$calcium release test/control ratios $\geq 1.3$. Differences in pH between test and control culture media were small (usually $<0.1$). No consistent association was established between *in vitro* osteolysis and a tumour's site, size, degree of differentiation or the presence of bone invasion or lymph node metastases.

Indomethacin was added to 14 of the co-culture experiments; the concentration used ($1\ \mu gml^{-1}$) has been shown to suppress prostaglandin synthesis to insignificant levels in this assay ($<1\ ngml^{-1}$)—see Tsao (1982). The results are included in Table I. Osteolysis was blocked to varying degrees by indomethacin and, in 10 tumours (71%), the level of inhibition was near to or greater than 50% of the total osteolytic activity. Inhibition was statistically significant in 6 tumours ($P<0.05$, Student's *t*-test). This consistent but incomplete blocking of tumour-associated bone resorption by indomethacin suggests that both prostaglandins and indomethacin-resistant (non-prostaglandin) osteolysins are produced.

In order to show that indomethacin affected the synthesis of new osteolytic factors rather than the action of osteolytic factors already released into the medium, the drug was also added to 3 tumour-conditioned media *after* instead of *during* the incubation period with tumour. Osteolysis was only slightly affected, confirming that indomethacin acts mainly by inhibiting prostaglandin formation.

*In vitro osteolysis and prostaglandin release in tumour-conditioned media* Synchronous observations on *in vitro* osteolysis and prostaglandin release were made with tumour-conditioned media (3 days incubation) from a further 12 tumours. The results are shown in Table II. Varying degrees of bone resorption were detected. Prostaglandins, especially $PGE_2$, were demonstrated in the conditioned media but the levels varied widely (42–120 $ngml^{-1}$, mean ±s.d. = 32.9±36.7 $ngml^{-1}$) and bore no direct relationship to the osteolysis observed. The amount of $PGE_2$ required to stimulate *in vitro* bone resorption in this system is ~5 to 10 $ngml^{-1}$ (Tsao, 1982) so the $PGE_2$ detected in the tumour-

**Table II** *In vitro* osteolysis and prostaglandin release in pre-conditioned media from 12 freshly-excised squamous carcinomas of the head and neck

| *Site of primary tumour* | *$^{45}Ca$ release: test/control ratio* | *Prostaglandins ($ngml^{-1}$)* | |
|---|---|---|---|
| | | *$PGE_2$* | *$PGF_{2\alpha}$* |
| Larynx | 2.80±0.10 | 42.6 | 5.0 |
| Tongue[a] | 2.61±0.19 | 12.0 | 0.8 |
| Larynx | 2.58±0.10 | 93.6 | 3.5 |
| Larynx | 2.56±0.07 | 15.1 | 12.9 |
| Tongue | 2.55±0.14 | 12.6 | 10.1 |
| Tongue[a] | 2.53±0.36 | 19.3 | 2.1 |
| Hypopharynx | 2.40±0.16 | 13.0 | 3.2 |
| Tongue | 2.33±0.08 | 4.2 | 4.3 |
| Hypopharynx | 2.30±0.02 | 25.6 | 3.8 |
| Maxillary antrum[b] | 2.25±0.06 | 120.0 | 3.2 |
| Larynx | 2.12±0.25 | 32.8 | 5.3 |
| Tongue[b] | 1.70±0.09 | 4.5 | 0.2 |
| | | Mean ±s.d. 32.9±36.7 ($n$=12) | Mean ±s.d. 4.5±3.6 ($n$=12) |

[a]nodal metastases.
[b]bone invasion demonstrated in surgical specimen.

conditioned media would account for at least part of the osteolytic activity demonstrated. $PGF_{2\alpha}$ was present in only small amounts and is unlikely to stimulate bone resorption to any extent.

Histological structure was well-preserved in the tumours after 3 days co-culture with calvaria. All the fragments contained intact carcinoma. Foci of necrosis, inflammation and fibrosis were also present.

*Observations with control tissues* Two sets of control tissues were examined: uninvolved tissues from the head and neck removed from surgical specimens at the same time as the tumours, and normal skin from reduction mammaplasties.

*Tissues from the head and neck* Osteolytic activity in 12 paired tumours and non-neoplastic tissues is shown in Table III. Ten of the control tissues resorbed bone *in vitro*. Activity was usually greater in the corresponding tumour, but the differences were small and reached statistical significance in only one instance ($P<0.01$, Student's $t$-test). Prostaglandins were measured in conditioned media from 4 of the pairs. Similar levels of $PGE_2$ were found in tumour and control tissues in 3 of the 4 pairs, the fourth showing an excess of $PGE_2$ in the tumour. Levels of $PGF_{2\alpha}$ followed no consistent pattern in the material examined. Sections from the cultured tissues showed no evidence of tumour but a variable amount of focal necrosis and mixed inflammatory infiltrates with mononuclear cells, lymphocytes and sometimes polymorphs.

*Normal skin In vitro* bone resorption and prostaglandin release were examined in 4 specimens of breast skin. Osteolysis was either weak or absent ($^{45}$Calcium release: test/control ratios of $0.87\pm0.02$, $1.10\pm0.04$, $1.48\pm0.14$, $1.47\pm0.10$). The amounts of prostaglandin detected were too low to stimulate bone resorption ($PGE_2<2.9\,ngml^{-1}$ and $PGF_{2\alpha}<1.3\,ngml^{-1}$). The cultured breast skin was histologically normal.

### *Observations with carcinoma cell lines*

In vitro *osteolysis* Eleven carcinoma cell lines were tested for bone resorbing activity using conditioned culture media (24 h incubation) obtained from subconfluent monolayer cell cultures. The results are shown in Table IV. Varying degrees of osteolysis were detected. Cell lines LICR/HN 2, 4, 6, 7, 10, 12 and 13 were moderately active and LICR/HN 1, 3, 5, and 9 were less active. The variations in activity noted among individual cell lines in repeated assays probably reflect variations in culture conditions such as cell density and batches of serum used. The pH differences between the test and control media were low (<0.1).

Indomethacin ($1\,\mu gml^{-1}$) was added to cultures of five of the active cell lines (LICR/HN 2, 4, 6, 7 and 12). Control medium contained the same amount of indomethacin. The results are shown in Table IV. In contrast to the findings with freshly

**Table III** *In vitro* osteolysis and prostaglandin release by paired neoplastic and non-neoplastic tissues

| *Tissue* | *$^{45}$Ca release test/control ratio* | *Prostaglandins ($ngml^{-1}$) $PGE_2$* | *$PGF_{2\alpha}$* |
|---|---|---|---|
| A) Bone resorption in co-culture experiments: | | | |
| Tumour (Floor of mouth) | $2.06\pm0.23$ | | |
| Control | $1.89\pm0.12$ | | |
| Tumour (Nasal septum) | $1.94\pm0.08$ | | |
| Control | $1.74\pm0.12$ | | |
| Tumour (Floor of mouth)[b] | $1.8\pm0.13$ | | |
| Control | $1.18\pm0.04$ | | |
| Tumour (Tongue) | $1.58\pm0.13$ | | |
| Control | $1.44\pm0.08$ | | |
| Tumour (Hypopharynx) | $1.5\pm0.11$ | | |
| Control | $1.48\pm0.16$ | | |
| Tumour[a] (Larynx) | $1.19\pm0.02$ | | |
| Control | $1.0\pm0.04$ | | |
| Tumour (Hypopharynx) | $1.19\pm0.08$ | | |
| Control | $1.28\pm0.08$ | | |
| Tumour (Tongue) | $1.08\pm0.03$ | | |
| Control | $1.22\pm0.23$ | | |
| B) Bone resorption and prostaglandin release in conditioned media: | | | |
| Tumour (Larynx) | $2.58\pm0.10$ | 93.6 | 3.5 |
| Control | $2.14\pm0.14$ | 96.4 | 10.9 |
| Tumour (Larynx) | $2.55\pm0.07$ | 15.1 | 12.9 |
| Control | $2.45\pm0.21$ | 14.0 | 2.4 |
| Tumour (Tongue) | $2.55\pm0.14$ | 12.6 | 10.1 |
| Control | $0.90\pm0.03$ | 1.4 | 1.5 |
| Tumour (Hypopharynx) | $2.40\pm0.16$ | 13.0 | 3.2 |
| Control | $2.68\pm0.16$ | 17.2 | 5.2 |

[a]nodal metastases.
[b]$P<0.01$ by Student's $t$-test.

**Table IV** *In vitro* osteolysis by 11 tumour cell lines derived from squamous carcinomas of the head and neck

| *Cell lines* | *Passage number* | *Cell density* ($\times 10^5 ml^{-1}$) | *Indo-methacin* ($1 \mu g ml^{-1}$) | $^{45}Ca$ *release (test/control)* |
|---|---|---|---|---|
| LICR/HN 6 | 20 | 2.9 | – | 1.87±0.14 |
| (Tongue) | 29 | | – | 1.83±0.13 |
| | 26 | 3.4 | – | 2.02±0.22 |
| | 26 | 3.6 | + | 2.19±0.15 |
| LICR/HN 4[a] | 16 | 2.2 | – | 1.60±0.06 |
| (Larynx) | | | | |
| | 24 | 1.9 | – | 2.21±0.10 |
| | 24 | 2.3 | + | 2.18±0.10 |
| LICR/HN 7 | 24 | | – | 1.75±0.07 |
| (Tongue) | | | | |
| | 21 | | – | 1.50±0.06 |
| | | | + | 1.68±0.02 |
| LICR/HN 13 | 7 | | – | 1.64±0.07 |
| (Oropharynx) | | | | |
| LICR/HN 10[a] | 4 | 2.7 | – | 1.61±0.05 |
| (Larynx) | | | | |
| LICR/HN 2[a] | 32 | 9.5 | – | 1.50±0.08 |
| (Larynx) | | | | |
| | 33 | 11.5 | – | 1.55±0.09 |
| | 33 | 9.4 | + | 1.30±0.04 |
| LICR/HN 12 | 21 | | – | 1.50±0.09 |
| (Hypopharynx) | | | + | 1.34±0.05 |
| LICR/HN 1 | 14 | 1.9 | – | 1.35±0.14 |
| (Tongue) | 17 | 1.9 | – | 1.52±0.13 |
| | 20 | | – | 1.31±0.03 |
| LICR/HN 5 | 48 | 2.6 | – | 1.28±0.03 |
| (Tongue) | 52 | | – | 1.32±0.02 |
| LICR/HN 9 | 7 | | – | 1.21±0.09 |
| (Tongue) | 15 | | – | 1.20±0.07 |
| LICR/HN 3 | 34 | | – | 0.89±0.04 |
| (Tongue) | 39 | | – | 1.27±0.07 |

[a]Bone invasion observed in original surgical specimens.

excised tumours (cf. Table I), no consistent effects were observed. The reduced osteolysis seen with cell lines LICR/HN 2 and 12 is not statistically significant.

*Radioimmunoassay of prostaglandins* In 5 carcinoma cell lines (LICR/HN 1, 3, 4, 5 and 6), prostaglandin concentrations and osteolytic activities were determined in the same culture medium. The results are shown in Table V. The concentrations of prostaglandins detected were low ($PGE_2 < 3.5\,ng ml^{-1}$, $PGF_{2\alpha} < 1.3\,ng ml^{-1}$) and did not correlate with the levels of osteolytic activity observed.

**Table V** Comparison of *in vitro* osteolysis and levels of prostaglandins $E_2$ and $F_{2\alpha}$ in cell culture supernatants from 5 squamous carcinoma cell lines

| *Cell line* | *Passage number* | $^{45}Ca$ *release (test/control)* | *Prostaglandins* ($ng ml^{-1}$) $PGE_2$ | $PGF_{2\alpha}$ |
|---|---|---|---|---|
| LICR/HN 6 (Tongue) | 29 | 1.83±0.13 | 0.7 | 0.21 |
| LICR/HN 4 (Tongue) | 16 | 1.60±0.06 | 3.3 | 0.2 |
| LICR/HN 5 (Tongue) | 52 | 1.32±0.02 | 0.7 | 0.22 |
| LICR/HN 1 (Tongue) | 20 | 1.31±0.03 | 1.5 | 0.2 |
| LICR/HN 3 (Tongue) | 39 | 1.27±0.07 | 1.4 | 1.3 |

Radioimmunoassay of prostaglandins was extended to the supernatant culture media collected from other tumour cell lines obtained under the same conditions as the bone resorption assay. The mean concentrations of prostaglandins detected in media from all 13 lines were generally low ($PGE_2$: $3.3 \pm 2.7\,ng ml^{-1}$, $PGF_{2\alpha}$: $0.25 \pm 0.21\,ng ml^{-1}$) though 3 cell lines (LICR/HN 7, 10 and 13) released higher amounts of $PGE_2$ ($6.5$–$8.8\,ng ml^{-1}$); these values are just significant in stimulating bone resorption *in vitro* (Tsao, 1982) and may account for a small proportion of the osteolytic activities previously observed.

Histological changes were examined in paired test and control calvaria from experiments with tumour cell lines LICR/HN 1, 2, 4 and 6. All slides were coded beforehand, but differences between the two groups were readily apparent. Bone incubated in control media felt firm when handled, and sections subsequently showed smooth intact trabeculae with only occasional multinucleate osteoclasts. Bones incubated with media from the carcinoma cell lines felt soft and sections showed a loss of bone substance with thin and irregularly outlined trabeculae and increased numbers of osteoclasts on or near the internal bone surface. The osteoclastic response was most marked in calvaria exposed to media from tumour cell lines LICR/HN 4 and 6; these two lines showed high levels of *in vitro* osteolysis and produced predominantly non-prostaglandin osteolysins (see Table IV).

*Observations with control (fibroblastoid) cell cultures* Fibroblastoid cell lines were examined for *in vitro* osteolysis and release of prostaglandins.

The results are shown in Table VI. Osteolytic activity was detected in 4/8 cultures. Differences in pH between test and control media were insignificant (pH<0.01). Prostaglandins were determined in media from two osteolytically active lines (FB 1 and 6) and two inactive lines (FB 2 and 4). The concentrations detected were low ($PGE_2 < 2.0\,ngml^{-1}$; $PGF_{2\alpha} < 0.7\,ngml^{-1}$) and were too small to stimulate bone resorption *in vitro*.

**Table VI** *In vitro* osteolysis and prostaglandin release by fibroblastoid cell lines

| *Fibroblastoid cells* | *Passage number* | *Cell density* ($\times 10^5 ml^{-1}$) | $^{45}Ca$ *release* (*test control*) | *Prostaglandins* ($ngml^{-1}$) $PGE_2$ | $PGF_{2\alpha}$ |
|---|---|---|---|---|---|
| FB 1 | 2 | 2.2 | 1.61±0.04 | ND | ND |
| | 4 | | ND | 2.0 | 0.2 |
| FB 5 | 5 | | 1.60±0.23 | ND | ND |
| FB 6 | 7 | | ND | 1.5 | 0.23 |
| | 8 | | 1.58±0.14 | ND | ND |
| FB 7 | 3 | | 1.32±0.09 | ND | ND |
| F 4 | 2 | 2.2 | 1.11±0.01 | ND | ND |
| | 3 | | ND | 1.5 | 0.7 |
| FB 2 | 2 | 2.7 | 1.09±0.09 | ND | ND |
| | 3 | | ND | 1.5 | ND |
| FB 8 | 5 | | 0.90±0.05 | ND | ND |
| FB 3 | 2 | 1.0 | 0.85±0.05 | ND | ND |

ND = not done.

### Discussion

Three main groups of findings have emerged from this work. *Freshly excised* squamous carcinomas of the head and neck resorb bone *in vitro*. Osteolytic activity, mediated by local osteoclasts, is consistently reduced (though not abolished) by indomethacin. The tumours release $E_2$ prostaglandins ($PGE_2$): the levels are variable but are sufficient to account for at least 50% of the bone resorption observed in most instances. *Cell lines* derived from squamous cancers are also osteolytic *in vitro* but differ from fresh tumours in two respects. Bone resorption by most of the cell lines is largely unaffected by indomethacin and production of $PGE_2$ is low, suggesting that prostaglandins are mainly implicated in osteolysis by fresh tumours. No differences in activity were observed between tumours treated pre-operatively by irradiation and/or cytotoxic drugs and tumours treated by primary surgery. *Non-neoplastic tissues* show a variable capacity to resorb bone *in vitro*, indicating that osteolysis is not a tumour-specific activity.

Several human tumours produce prostaglandins *in vitro*, notably carcinomas of the breast (Bennet *et al.*, 1975, 1976, 1977*a*; Powles *et al.*, 1976; Dowsett *et al.*, 1976; Greaves *et al.*, 1980) kidney (Atkins *et al.*, 1977) and large intestine (Bennett *et al.*, 1977*b*). Prostaglandins from human tumours resorb bone *in vitro*, and extensive studies of prostaglandin-mediated osteolysis have been made with experimental tumours (Tashjian *et al.*, 1972, 1982; Voelkel *et al.*, 1978; Tashjian, 1978). Activation of local osteoclasts has been observed in the *in vitro* model systems (Schelling *et al.*, 1980). Investigators have been unable to establish a linear relationship between levels of prostaglandins released and the mass of bone resorbed, and it has become apparent that additional non-prostaglandin osteolytic factors are also involved. Particular discussion continues in relation to two topics—the cellular origin of the osteolysins and their nature, especially with respect to non-prostaglandin substances.

(1) The complementary use of fresh tissues and cell lines provides a starting point for separating tumour and host cells as potential sources of osteolytic agents. In the experiments described here with paired tumour and control tissues, most of the fresh control tissues resorbed bone *in vitro* and released prostaglandins at levels not significantly less than the corresponding tumour. The absence of tumour in control tissues was always confirmed histologically, but they were invariably inflamed and had usually been exposed to pre-operative irradiation and/or chemotherapy; although such tissues form acceptable controls for the tumours from the same patients, they clearly cannot be regarded as normal. By contrast, histologically normal breast tissue with no necrosis or inflammatory infiltrates showed negligible osteolysis and prostaglandin release. Mononuclear macrophages can synthesise prostaglandins and resorb bone *in vitro* (Mynett *et al.*, 1975; Humes *et al.*, 1977; Mundy *et al.*, 1977; Kahn *et al.*, 1978; McArthur *et al.*, 1980) and there is evidence that bone resorption in rheumatoid arthritis and peridontal inflammation may be partly due to local production of prostaglandins (Robinson *et al.*, 1975; Harris, 1978). Some of the control fibroblastoid cell lines resorbed bone *in vitro* but $PGE_2$ levels were always low. Taken together, these findings suggest an association between the capacity of non-neoplastic tissues to manifest prostaglandin-mediated bone resorption *in vitro* and the presence of inflammatory cells within them. This is a difficult topic to pursue in intact tissues as the inflammatory infiltrates would need to be quantified and each of the cell constituents accurately identified.

(2) The nature of non-prostaglandin

(indomethacin-insensitive) osteolysins remains obscure. Candidates include ectopic parathyroid hormone, osteoclast activating factors and certain other ill-defined products (Mundy *et al.*, 1974*a, b*; Josse *et al.*, 1981; Nimberg *et al.*, 1982). The non-prostaglandin osteolysin associated with squamous cancers of the head and neck is uncharacterized at the present time.

No consistent relationship has emerged between bone resorbing activity and prostaglandin release *in vitro*, clinicopathological features of the tumours (including the presence of bone invasion in the surgical specimens) and post-operative survival of the patients. A similar lack of correlation has been reported in patients with breast cancer (Dady *et al.*, 1981) though prognostic significance for raised prostaglandin levels has been claimed by other workers (Fitzpatrick & Stringfellow, 1979).

We are deeply indebted to Drs. G.C. and D.M. Easty for their invaluable help in the early phases of this work, and to Mr. H.J. Shaw, Dr. V.M. Dalley and Mr. P. Clifford for access to their patients.

Financial support is acknowledged from the Shell Company (Hong Kong) Ltd. (S-WT), the Medical Research Council (JFB, RLC) and the Vandervell Foundation (MRP).

### References

ATKINS, D., IBBOTSON, K.J., HILLER, K., HUNT, N.H., HAMMONDS, J.C. & MARTIN, T.J. (1977). Secretion of prostaglandins as bone-resorbing agents by renal cortical carcinoma in culture. *Br. J. Cancer*, **36**, 601.

BENNETT, A., McDONALD, A.M., SIMPSON, J.S. & STAMFORD, I.F. (1975). Breast cancer, prostaglandins and bone metastases. *Lancet*, **i**, 1218.

BENNETT, A., CHARLIER, E.M., McDONALD, A.M., SIMPSON, J.S. & STAMFORD, I.F. (1976). Bone destruction by breast tumours. *Prostaglandins*, **11**, 461.

BENNETT, A., CHARLIER, E.M., McDONALD, A.M., SIMPSON, J.S., STAMFORD, I.F. & ZEBRO, T. (1977*a*). Prostaglandins and breast cancer. *Lancet*, **ii**, 624.

BENNETT, A., DEL TACCA, M., STAMFORD, I.F. & ZEBRO, T. (1977*b*). Prostaglandins from tumours of human large bowel. *Br. J. Cancer*, **35**, 881.

BENNETTT, A., CARTER, R.L., STAMFORD, I.F. & TANNER, N.S.B. (1980). Prostaglandin-like material extracted from squamous carcinomas of the head and neck. *Br. J. Cancer*, **41**, 204.

CARTER, R.L. (1982). Morphological patterns of bone destruction by infiltrating tumours. In *Prostaglandins and Cancer: First International Conference*. (Eds. Powles *et al.*) Alan R. Liss, New York, p. 561.

DADY, P.J., POWLES, T.J., DOWSETT, M., EASTY, G., WILLIAMS, J. & NEVILLE, A.M. (1981). *In vitro* osteolytic activity of human breast carcinoma tissue and prognosis. *Br. J. Cancer*, **43**, 222.

DOWSETT, M., EASTY, G.C., POWLES, T.J., EASTY, D.M. & NEVILLE, A.M. (1976). Human breast tumour-induced osteolysis and prostaglandins. *Prostaglandins*, **11**, 447.

EASTMAN, A.R. & DOWSETT, M. (1976). The simultaneous separation of individual prostaglandins by thin layer chromatography on an unmodified support. *J. Chromatogr.*, **128**, 224.

EASTY, D.M., EASTY, G.C., CARTER, R.L., MONAGHAN, P. & BUTLER, L.J. (1981*a*). Ten human carcinoma cell lines derived from squamous carcinomas of the head and neck. *Br. J. Cancer*, **43**, 772.

EASTY, D.M., EASTY, G.C., CARTER, R.L., MONAGHAN, PITTAM, M.R. & JAMES, T. (1981*b*). Five human tumour cell lines derived from a primary squamous carcinoma of the tongue, two subsequent local recurrences and two nodal metastases. *Br. J. Cancer*, **44**, 363.

FITZPATRICK, F.A. & STRINGFELLOW, D.A. (1979). Prostaglandin D formation by malignant melanoma cells correlates inversely with cellular metastatic potential. *Proc. Natl Acad. Sci.*, **76**, 1765.

GREAVES, M., IBBOTSON, K.J., ATKINS, D. & MARTIN, T.J. (1980). Prostaglandins as mediator of bone resorption in renal and breast tumours. *Clin. Sci.*, **58**, 201.

HARRIS, M. (1978). Odontogenic cyst growth and prostaglandin-induced bone resorption. *Ann. R. Coll. Surg.*, **62**, 85.

HUMES, J.L., BONNEY, R.J., PELUS, L. & 4 others. (1977). Macrophages synthesise and release prostaglandins in response to inflammatory stimuli. *Nature*, **269**, 149.

JOSSE, R.G., MURRAY, T.M., MUNDY, G.R., TEZ, D. & HIRSCHE, J.N.H. (1981). Observations on the mechanisms of bone resorption induced by multiple myeloma marrow culture fluids and partially purified osteoclast-activating factor. *J. Clin. Invest.*, **67**, 1472.

KAHN, A.J., STEWART, C.C. & TEITELBAUM, S.L. (1978). Contact-mediated bone resorption by human monocytes *in vitro*. *Science*, **199**, 988.

McCARTHUR, W., YAARI, A.M. & SHAPIRO, I.M. (1980). Bone solubilization by mononuclear cells. *Lab. Invest.*, **42**, 450.

MUNDY, G.R., LUBEN, R.A., RAISZ, L.G., OPPENHEIM, J.J. & BUELL, D. (1974*a*). Bone resorbing activity in supernatants from lymphoid cell lines. *N. Engl. J. Med.*, **290**, 867.

MUNDY, G.R., RAISZ, L.G., COOPER, R.A., SCHECTER, G.P. & SALMON, S.E. (1974*b*). Evidence for the secretion of an osteoclast stimulating factor in myeloma. *N. Engl. J. Med.*, **291**, 1041.

MUNDY, G.R., ALTMAN, A.J., GONDEK, M.D. & BANDELIN, J.G. (1977). Direct resorption of bone by human monocytes. *Science*, **196**, 1109.

MYNETT, L., BRAY, M.A., GORDON, D. & MORLEY, J. (1975). Macrophages on intrauterine contraceptive devices produce prostaglandins. *Nature*, **257**, 227.

NIMBERG, R.B., HUMPHRIES, D.E., LLOYD, W.S., WELLS, M. & SCHMID, K. (1982). Purification and partial characterization of a protein from cancer ascites fluid which stimulates the resorption of bone explants *in vitro*. *J. Biol. Chem.*, **257**, 2477.

POWLES, T.J., DOWSETT, M., EASTY, D.M., EASTY, G.C. & NEVILLE, A.M. (1976). Breast cancer osteolysis, bone metastases and antiosteolytic effects of aspirin. *Lancet*, **i**, 608.

REYNOLDS, J.J. (1968). Inhibition by calcitonin of bone resorption induced *in vitro* by vitamin A. *Proc. R. Soc. B.*, **170**, 61.

ROBINSON, D.R., TASHJIAN, A.H. & LEVINE, L. (1975). Prostaglandin-stimulated bone resorption by rheumatoid synovia. *J. Clin. Invest.*, **56**, 1181.

SCHELLING, S.M., WOLFE, H.J. & TASHJIAN, A.H. (1980). Role of the osteoclast in prostaglandin $E_2$-stimulated bone resorption. *Lab. Invest.*, **42**, 290.

TASHJIAN, A.H. Jr. (1978). Role of prostaglandins in the production of hypercalcemia by tumors. *Cancer Res.*, **38**, 4138.

TASHJIAN, A.H., VOELKEL, E.F., LEVINE, L. & GOLDHABER, P. (1972). Evidence that the bone resorption-stimulating factor produced by mouse fibrosarcoma cells is prostaglandin $E_2$. *J. Exp. Med.*, **136**, 1329.

TASHJIAN, A.H. Jr., VOELKEL, E.F. & LEVINE, L. (1982). Prostaglandins, tumor cells and bone metabolism. In *Prostaglandin and Cancer: First International Conference* (Eds. Powles *et al.*) Alan R. Liss, New York, p. 513.

TSAO, S.-W., BURMAN, J.F., EASTY, D.M., EASTY, G.C. & CARTER, R.L. (1981). Some mechanisms of local bone destruction by squamous carcinomas of the head and neck. *Br. J. Cancer*, **43**, 392.

TSAO, S.-W. (1982). *Mechanisms of Bone Destruction by Squamous Carcinomas of the Head and Neck*. Ph.D. Thesis. University of London.

TSAO, S.-W., BURMAN, J.F. & CARTER, R.L. (1983). Hypercalcaemia and *in vitro* osteolysis associated with xenografts of squamous carcinomas of the tongue. *Br. J. Cancer*, **48**, 103.

VOELKEL, E.F., TASHJIAN, A.H., FRANKLIN, R., WASSERMAN, E. & LEVINE, L. (1975). Hypercalcemia and tumor prostaglandins: The $VX_2$ carcinoma model in the rabbit. *Metabolism*, **24**, 973.